# Stem Cell Hierarchy and Clonal Evolution in Acute Lymphoblastic Leukemia

**DOI:** 10.1155/2015/137164

**Published:** 2015-07-06

**Authors:** Fabian Lang, Bartosch Wojcik, Michael A. Rieger

**Affiliations:** ^1^Department of Hematology/Oncology, Goethe University Hospital, Theodor-Stern-Kai 7, 60590 Frankfurt am Main, Germany; ^2^LOEWE Center for Cell and Gene Therapy Frankfurt, Goethe University, Theodor-Stern-Kai 7, 60590 Frankfurt am Main, Germany; ^3^German Cancer Consortium (DKTK), Heidelberg, Germany; ^4^German Cancer Research Center (DKFZ), Im Neuenheimer Feld 280, 69120 Heidelberg, Germany

## Abstract

Cancer is characterized by a remarkable intertumoral, intratumoral, and cellular heterogeneity that might be explained by the cancer stem cell (CSC) and/or the clonal evolution models. CSCs have the ability to generate all different cells of a tumor and to reinitiate the disease after remission. In the clonal evolution model, a consecutive accumulation of mutations starting in a single cell results in competitive growth of subclones with divergent fitness in either a linear or a branching succession. Acute lymphoblastic leukemia (ALL) is a highly malignant cancer of the lymphoid system in the bone marrow with a dismal prognosis after relapse. However, stabile phenotypes and functional data of CSCs in ALL, the so-called leukemia-initiating cells (LICs), are highly controversial and the question remains whether there is evidence for their existence. This review discusses the concepts of CSCs and clonal evolution in respect to LICs mainly in B-ALL and sheds light onto the technical controversies in LIC isolation and evaluation. These aspects are important for the development of strategies to eradicate cells with LIC capacity. Common properties of LICs within different subclones need to be defined for future ALL diagnostics, treatment, and disease monitoring to improve the patients' outcome in ALL.

## 1. Introduction

Fundamental evidence has evolved over the last decades showing that tumors are not of a homogeneous cell composition but are comprised of a mixture of immature stem/progenitor cells and more differentiated cells. Tumors thereby resemble the organization of normal tissue. Considerable heterogeneity exists between individual patients suffering from the same cancer type (intertumoral heterogeneity), between subpopulations of the same tumor (subclonal heterogeneity) and even between cells of the same subpopulation (cellular heterogeneity) [1–5]. Different events may contribute to the observed heterogeneity: two models have been postulated that may explain heterogeneity: first, the cancer stem cell (CSC) model [6] and, second, the clonal evolution model [7]. The CSC model describes a hierarchical organization of tumor cell subpopulations with most immature stem cell-like CSCs at the apex of a malignant differentiation hierarchy. The hierarchy can be steep with only rare CSCs giving rise to more differentiated, non-tumor-propagating cells, or flat with many CSCs and only some differentiated tumor cells. In contrast, in the clonal evolution model, the successive accumulation of genetic alterations in distinct cells dictates the appearance and growth of subclones. There is no ordered hierarchy of distinct subclones. Importantly, both models might not be mutually exclusive and a combination of both models is probably resembled in most tumors. The consideration of the heterogeneity has clinical implications, as it might be the underlying reason for therapeutic failure, treatment resistance, and relapse. There is a broad interest in the identification of CSCs in solid tumors as well as in hematologic malignancies. This also holds true for acute lymphoblastic leukemia (ALL); however, the existence, the phenotype, and the biology of CSCs, the so-called leukemia-initiating cells (LICs), remain controversial [8]. ALL is a highly malignant cancer of lymphoid progenitor cells in the bone marrow, which is characterized by the uncontrolled expansion of leukemic blasts. ALL can be divided into different subtypes determined by age (adult versus pediatric), lineage origin (T- versus B-ALL), immunologic findings (pro-, pre-, common, and mature B-ALL, resp. early, thymic, and mature T-ALL), and genetic findings (i.e.,* BCR-ABL* positive or negative) [9]. Using these parameters, ALLs are grouped into risk categories, with an average 5 years' survival of 35% taking all risk groups together [10–12].

Analysis of the heterogeneity of ALL cells and of the temporal changes of the subclonal architecture has provided insights into the dynamics and hierarchical relationship of leukemic clones that develop during the clinical course of the disease and evolve resistance to therapy [13]. However, unraveling the regulatory mechanism controlling the biological characteristics of LICs, for example, self-renewal, proliferative capacity, or antiapoptotic machinery, should provide clinically relevant information on novel molecular targets and treatment strategies. The clinical relevance of such approaches is vital for relapsed or refractory ALL, which is associated with a dismal outcome and long-term survival of less than 10% [10–12].

In this review, we discuss the concepts of stem cell hierarchy and clonal evolution in their appliance to B-ALL and shed light on major controversies and obstacles in LIC research in this entity.

## 2. The Cancer Stem Cell Concept

### 2.1. Definition

CSCs are defined as cells within a tumor that have the unique ability to self-renew, reinitiate the disease, and reconstitute all different tumor cells. Therefore, CSCs stand at the apex of a tumor cell hierarchy. They resemble functional similarities to normal somatic stem cells, that is, hematopoietic stem cells (HSCs) with their capacity to renew themselves and to give rise to all mature blood cell lineages [14, 15].

A common terminology for cells with specific properties in ALL used in this review should be introduced: the leukemic cell of origin (LCO) is the first cell carrying the initial preleukemic lesion. This event occurred during normal hematopoiesis and will finally pave the way for disease initiation later on. LICs (or tumor-initiating cells (TICs) in solid tumors) are cells which initiate and maintain the disease. They are defined by their functional capacity to initiate leukemia in a mouse transplantation model* in vivo*. These cells are also called leukemic stem cells (LSCs). LSCs in leukemia, and CSCs in solid cancers, are terms that emerged by their molecular and functional similarities to their somatic stem cell counterparts. However, it also infers that LSCs arise directly from their stem cell counterparts, which is not necessarily the case and also no prerequisite to acquire stem cell-like functions [16, 17].

We preferentially use the term LICs rather than LSCs in this review for cells that have stem-cell like features and can reinitiate the full-blown disease* in vivo*, which can only be read out experimentally by leukemia induction in immune-deficient mice. The functional abilities of LICs to initiate and maintain the disease, and probably also giving rise to relapse, make these cells a prime target for rational therapy developments.

### 2.2. History

In 1937, Furth et al. showed that a tumor can arise from a single cell [18]. By injecting limited cell dilutions of lymphoid and myelogenous leukemia cell lines up to a single cell into mice, they demonstrated that less than 5% of cells were capable of inducing leukemia. In the 1950s, transplantation experiments of solid tumor cells revealed on rare occasions the successful transduction of the disease by single cell inoculation of rat Yoshida sarcoma [19], mouse sarcoma [20], or rat ascites tumor [21]. In 1963, Bruce and van der Gaag reported that less than 1% of mouse lymphoma cells could give rise to spleen colonies, which were also capable of inducing lymphoma upon serial transplantation [22]. A new major topic in cancer research for a broad variety of malignancies was launched in the mid-1990s when Lapidot et al., Blair et al., and Bonnet and Dick prospectively identified a LIC phenotype in acute myeloid leukemia (AML) [23–25].

### 2.3. Solid Tumors

During the last decade, TICs have been functionally identified in many solid cancer entities: in breast, brain, ovarian, prostate, colon, pancreatic, hepatic, gastric, lung cancer, and melanoma [26–35]. Despite these enormous achievements in CSC research, further progress is clearly hindered by the lack of robust markers for prospective identification of TICs in distinct tumor types. Surface markers and functional properties distinctive for TICs have been used to enrich for them: that is, surface markers CD24, CD44, and CD133 and the multidrug efflux efficacy are commonly integrated in TIC isolation procedures [26, 27, 31, 36–38]. However, many of these markers are controversial in the field, as opposing reports demonstrate equal TIC activity in marker-negative populations [31, 36, 39, 40]. Therefore, robust markers for TICs in solid cancers are urgently awaited.

### 2.4. Hematologic Malignancies

The best characterized hematologic malignancies in regard to LICs are acute and chronic myeloid leukemia (AML and CML, resp.) [41, 42]. AML was the first malignancy with a reported distinct surface marker phenotype for LICs [24, 25]. Bonnet and Dick described the existence of a small fraction of cells within the leukemic bulk (0.2–200 per 1 × 10^6^ leukemic cells) that solely had the capacity to repopulate the entire disease after transplantations in immunocompromised mice and to self-renew and differentiate upon serial transplantations [25]. These cells were found exclusively in the CD34+CD38−Lin− compartment. Importantly, the same marker-defined compartment also contains normal long-term repopulating hematopoietic stem cells. They further explored cells with a reduced repopulation ability at different stages of differentiation that resembled a leukemic hierarchy with the LIC at the apex, also alike the hematopoietic hierarchy of normal HSCs [43]. Blair et al. identified a similar CD34− expressing LIC phenotype in AML that could be distinguished from normal HSCs by their lack of CD90 expression [24]. CML is a molecularly well-characterized disease driven by the translocation product* BCR-ABL *(“Philadelphia chromosome”, Ph). In experimental murine models of CML, BCR-ABL expression initiated the disease only in immature stem and progenitor cells (LSK cells), but not in committed myeloid progenitors [44, 45]. It has been repetitively confirmed that these BCR-ABL+ LSK cells are exclusively capable of inducing CML in secondary recipients in a dose dependent manner [46, 47]. The LIC fraction in CML has been intensively studied to identify LIC-specific features, pathways, and targets for novel stem cell-directed therapies in CML (reviewed by Zhang and Li) [48].

### 2.5. Similarities between LICs and HSCs

HSCs maintain hematopoiesis life-long by their multipotency and self-renewal [49]. These rare cells (about 0.01–0.2% of total mononuclear bone marrow cells in humans) stand at the apex of a differentiation hierarchy to give rise to highly proliferative multipotent (MPPs) and lineage-restricted progenitors (e.g., granulocyte-macrophage progenitors and megakaryocyte-erythrocyte progenitors) and finally to all mature blood cells [49]. Since HSCs are largely quiescent, their genomic integrity is preserved, and frequent replications can introduce DNA mutations and may lead to oncogenic transformation [50]. The quiescent state also protects the HSCs from exhaustion. They primarily divide in hematopoietic stress situations, such as blood loss or infections [51, 52]. HSCs reside in specific niches in the bone marrow and their function is dependent on a complex interplay of cell extrinsic and intrinsic factors governing HSC fate decisions [49]. The niche dependency for HSC self-renewal becomes drastically obvious when HSCs are taken in culture where they spontaneously start to differentiate in the absence of their niche support.

LICs may be seen as the malignant functional counter part of HSCs, standing at the apex of a leukemic differentiation hierarchy. In fact, LICs and HSCs share many functional and molecular features. LICs are able to initiate and maintain the disease due to their self-renewal ability. They produce more differentiated leukemic progeny, so called leukemic blasts, which are highly proliferative, have a block in terminal differentiation and defects in apoptosis mechanisms [53, 54]. LICs often share surface marker combinations (e.g., CD34+CD38−Lin−) that also appear on human HSCs. Abilities like quiescence, increased efflux pump activity and localization in distinct bone marrow niches, comparable to the HSCs, making the LICs rather resistant to various standard therapies [55, 56]. This considerable overlap of functional and molecular features should not imply that LICs necessarily originate from HSCs that received the first transforming mutations, but one should emphasize that LICs have acquired functional and molecular features of stem cells that clearly provide a selective benefit. A major goal is to determine functional, molecular, and biochemical differences between LICs and HSCs to develop LIC-specific agents for therapy and diagnostics. LICs also reside in the bone marrow as do HSCs. It is currently debated whether LICs occupy the same niche as HSCs, whether they are more niche independent or whether they can even shape their own leukemic niche. The appealing concept emerged that LICs reprogram the bone marrow niche according to their needs from studies on myeloid dysplastic syndrome [57].

## 3. LICs in B-ALL

In B-ALL, robust and stabile phenotypes and functional data of LICs and their respective leukemic differentiation hierarchy are highly controversial (Table 1). B-ALLs were considered as malignant counterparts of the developmental hierarchy of normal B cell with CD34+ CD19− stem/progenitor cells at the apex of B cell development [58, 59]. Indeed, originally the compartment of CD34+ CD19− cells was reported to contain LICs exclusively [60], which was confirmed in several subsequent studies [58, 61, 62]. Later, CD19 was identified as a LIC-specific marker in standard and high risk ALL [59, 63]. Furthermore, it was demonstrated, that self-renewal was not restricted to CD34+ CD19− cells exclusively, but was also found in various populations with a large spectrum of developmental stages [64, 65]. Most important, it was confirmed that the majority of mature as well as immature ALL blasts can repopulate the entire disease [66]. These findings clearly challenge the stem cell concept in ALL or at least indicate a flat hierarchical organization. Conflicting results also emerged even for phenotypically identical B-ALLs as, for example, the absence and presence of CD10 was defined as LIC specific [61]. Along the same line, CD34+ and CD34− cells have the same leukemia initiation potential in infant MLL gene rearranged ALL [67]. The authors suggested CD9, CD32, and CD24 as more useful in enriching for LICs, however, only based on expression data [67]. CD34+ CD38− CD58− cells were postulated to be LIC-specific in BCR-ABL+ ALL [68]. Although no robust surface marker combination has been described yet to isolate LICs at high purity in a broad range of B-ALLs up to date, the search for specialized stem-cell like LICs in B-ALL might not be obsolete, and maybe the common surface markers known from the normal B cell developmental hierarchy might not be suitable. This further emphasizes the need to find new markers of LICs for their prospective identification.

However, the possible reasons for the reported discrepancies of B-ALL LIC-related markers must be carefully considered for future attempts to find better LIC markers. Cell plasticity in lymphoid cells may explain some of these opposing results: for example, B cells can be transdifferentiated into macrophages* in vivo* [69]; they can be differentiated to B cell phenotype tumors by the loss of Pax5, the B cell lineage commitment factor [70], and reprogrammed into pluripotent stem cells [71]. In concordance with this notion, the loss of the B cell differentiation phenotype in Hodgkin's lymphoma can cause the acquisition of stem cell properties [72, 73]. High plasticity is also proven in transformation from B cell lymphomas and chronic lymphocytic leukemias to histiocytic and dendritic sarcomas [74, 75]. Therefore, the remarkable phenotypical and transcriptional plasticity must be taken into consideration in the search for stable LIC markers in B-ALL. Other explanations for the conflicting results are the variety of different methods used to define LIC activity as well as the origin of patient material, its storage, preparation, and purification, as further eluted in Section 6: “Technical challenges.” Clearly, different B-ALL disease subtypes used in different studies reflect that B-ALLs are very heterogeneous [76]. Therefore, results in LIC evaluation differ and must be compared with caution: here, one has to mention adult versus pediatric B-ALLs, different immunophenotypes (common ALL, pre-B-ALL, pro-B-ALL, etc.), different genotypes, and mutational status (e.g., BCR-ABL positive versus negative, MLL rearrangement positive or negative) as also described in Table 1.

Another complicating factor is the ongoing evolution of the leukemic clones within ALL. ALL LICs are genetically heterogeneous. As multiple subclones with LIC potential evolve over time, a precise marker-defined identification of the LIC cell compartment becomes challenging. Moreover LIC properties at a given time point do not necessarily reflect the nature of the initial leukemic cell of origin due to ongoing clonal evolution [77].

Many intriguing questions remain in the field of LICs in ALL: is there evidence for LIC-driven stem cell-like hierarchy in ALL at all? There are some experimental cancer systems which do not adhere to a CSC model, as shown in mouse lymphoma [78, 79] or melanoma. Does leukemia engraftment in mouse really reflect the existence of LICs being responsible for disease maintenance, refraction, and relapse in human? Has the LIC in ALL a variable or high plastic phenotype and therefore a prospective identification is not possible at all?

Clearly, the established markers do not define LICs properly in B-ALL and taking into account the mentioned facts of LICs in ALL, future marker validation must be performed with caution.

## 4. Clonal Evolution in ALL

### 4.1. Definition

Clonal evolution and subclonal diversity are hallmarks of the pathophysiologic mechanisms of many cancers including ALL. A successive acquisition of genetic alterations dominates the clonal evolution. This clonal progression is an evolutionary process that is driven by selection and expansion of adapted subclones [80]. The evidence of intratumoral genetic heterogeneity has been initially provided by various techniques: chromosome karyotyping [1], genetic analysis of multifocal cancers [2], FISH (fluorescence in situ hybridization) based tissue section screening [3], immunophenotype based cell analysis [81], molecular probing of multiple small biopsies, microselected tissues [4], and sector ploidy profiling [5]. A fundamental improvement in subclonal discrimination has been made by copy number alteration (CNA) analysis and next generation deep parallel sequencing (NGS) of many cancer entities and leukemia types. These genome wide studies revealed many driver and passenger mutations in cancer reflecting also the genetic landscape of subclonal heterogeneity and intraclonal genetic diversity [82, 83]. The degree of genetic diversification has been linked to poorer prognosis in malignancies like breast and pancreatic cancer [84, 85].

### 4.2. Analyses of Monozygotic Twins and Paired Samples from Diagnosis and Relapse

Major insights in development and composition of subclonal architecture in ALL have been gained by Mel Greaves and his group analyzing ALL emergence and progression in monozygotic twins [86–90]. First, these studies revealed that the original ALL-specific mutations can already appear in utero and create a preleukemic subclonal compartment. These early subclones do not have the capability of disease induction. Later in life, distinct subclones gain full leukemic potential by the stepwise acquisition of subsequent mutations leading to ALL development. These additional alterations emerged independent from the original mutation of the ancestral clone, and many different subclones build a massive intra- and intertumoral heterogeneity.

The importance of additional mutations to create full leukemic potential in a preleukemic ancestral clone was supported by the analysis of a monozygotic twin pair with one healthy twin and one twin who developed ALL, albeit both twins carried the* BCR-ABL* fusion transcript. Interestingly, a common “stalled” preleukemic clone was detected even in the healthy twin [63, 88]. This aspect is also supported by the fact that 1% of newborns show ETV6-RUNX1 expression in the B cell lineage, but the incidence of ALL is 100 times smaller [89]. Furthermore, clonotypic immune globulin rearrangements and* ETV6-RUNX1* mutations were detected in the B lineage compartment also in healthy newborns that later developed disease [90]. In conclusion, heterogeneity in ALL has a prenatal in utero origin, with further mutations being required to establish subclones with full leukemic potential which later develop independently and build up massive subclonal heterogeneity.

Groundbreaking work in deciphering the dynamic clonal heterogeneity in paired samples of relapse and primary diagnosis from the same patient has been performed by Mullighan and colleagues [91, 92]. It has been revealed that the number of CNAs varies remarkably between primary diagnosis and relapse and in here, the number of CNAs increases in relapse. This reflects a change in subclones being present at primary diagnosis, after therapy and during relapse. However, the dominant subclone in relapse has already been present as a minor subclone at primary diagnosis [92]. This finding has been confirmed by deep NGS methods [93]. Therefore, leukemia progression is not associated with ongoing genetic instability within one homogeneous compartment but rather with clonal evolution and selection of a large, but limited number of subclones. Obviously, a minor subclonal population at diagnosis is resistant to therapy and therefore is able to escape therapy giving rise to relapse [92, 93]. It seems likely that ongoing clonal evolution as a major mechanism for heterogeneity seems to occur already in the preleukemic phase [94]. Furthermore, the presence of multiple relapse subclones has been confirmed [92, 94–97]. These relapse subclones partially showed significant changes in terms of surface marker expression and IgH locus patterns, which has significant consequences for the correct measurement of minimal residual disease in ALL patients [98].

### 4.3. Linear versus Branching Succession Model

**Figure 1:**
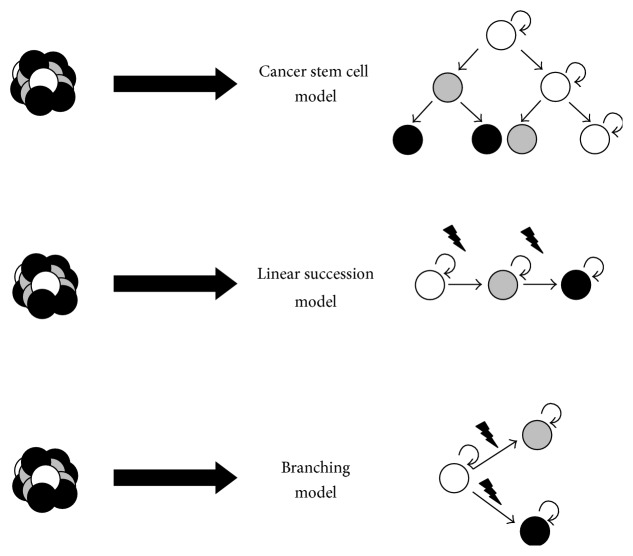
Different models explaining tumor cell heterogeneity. The cancer stem cell model describes stem cell-like LICs at the apex of a tumor cell differentiation hierarchy, exclusively having self-renewal potential and giving rise to all other cells of the leukemic bulk cells, which do not have LIC activity. In contrast, the clonal evolution models show no differentiation hierarchy and the main assumption is that individual subclones acquire successive mutations resulting in an ongoing subclonal evolution leading to intratumoral heterogeneity either in a linear or branching fashion. Importantly, these models are not mutually exclusive and a combination of both models contributes to tumor cell heterogeneity.

The clonal heterogeneity seems to be much more complex than originally anticipated. Gawad et al. revealed the different frequencies of subclones by single cell whole genome sequencing of 1500 single cells in childhood ALL cases [99]. This study describes the presence of more than 4000 different leukemic subclones within one patient being present at the same stage of disease, which confirmed the assumption of immense clonal heterogeneity. The development of clonal heterogeneity can be explained by two different models: the linear succession model and the branching evolution model (Figure 1). In the linear succession model, one clone acquires stepwise novel mutations. In here, subclones of different mutational stage do exist in parallel but are related in a linear genealogy. In the branching evolution model, on the contrary, subclones divide in a branching, nonlinear fashion. This results in subclones at different evolution levels which do exist in parallel. They have emerged from a common ancestral clone but are not directly related with each other.

**Figure 2:**
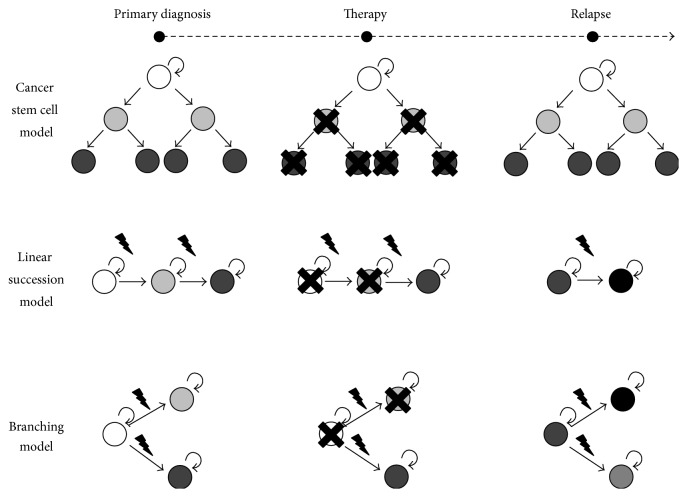
Treatment escape leading to relapse in the stem cell and clonal evolution models. In the cancer stem cell model, all leukemic bulk cells are eradicated by therapy, apart from stem cell-like LICs, which are resistant to therapy and give rise to relapse. In the clonal evolution model, distinct subclones acquire the capability of therapy resistance by ongoing mutations. Subclones that were not eradicated during therapy provide then the leukemic reservoir for relapse.

Originally, it was anticipated in cancer that clones develop in the linear succession model [100]. However, tracing distinct IgH rearrangements of individual subclones and their ancestral relationship revealed that the underlying clonal evolution model is rather highly branching and complex [101–103]. Also the relative clonal frequencies in ETV6-RUNX1 positive ALL cases recapitulated a picture of a very complex clonal architecture with up to 10 subclones interrelated via an ancestral branching tree even in one sample [104]. Similar findings have been made in MLL positive ALLs with high clonal variegation [105] and in therapy resistant Ph+ ALL cases [106]. Genome wide CNA analysis of paired diagnostic samples revealed that relapse subclones are rarely identical with dominant subclones at diagnosis. Therefore, they are derived from an ancestral clone and diverged early during tumorigenesis, with multiple subclones being identified, which are related in a complex branching rather than a linear evolution model [92, 98, 102, 104, 105, 107–111]. All these findings strongly hint for a branching Darwinian selection model that is the cause of ALL propagation, relapse, and clonal evolution (Figure 2). This is in line with findings in other cancers that are composed of a nonlinear branching subclonal architecture [112–114].

### 4.4. Influence of Therapy on Clonal Architecture

Another important aspect is the influence of therapy on the clonal architecture of the disease. The clonal evolution already begins at an early stage of the disease and multiple subclones are already established at primary diagnosis without any previous therapy. These subclones have different capabilities in terms of survival, proliferation, and therapy resistance and may compete with each other. Once the treatment has started, therapeutic pressure is added to the subclonal competition and those subclones being drug sensitive will be eradicated. This in turn leads to a survival benefit of drug resistant subclones which can outgrow after a certain delay and cause the relapse. After the eradication of the majority of subclones, the clonal evolution and competition within the remaining resistant subclones start again leading to a novel heterogeneous mixture of subclones (Figure 2). With every treatment round, developmental bottlenecks are created leading to a clonal selection process, which in the end results in the selection of highly drug resistant and aggressive subclones.

This dynamic clonal evolution has been demonstrated in chemoresponsive and chemoresistant ALL cases [108] and by the molecular composition of childhood ALL samples under chemotherapeutic drug pressure [115]. Variegated subclones could be artificially rendered by* in vivo* chemotreatment. Hereby, resistant subclones showed even higher CNA alterations, which may reflect high aggressive original samples [115]. Further analyses also revealed emerging mutations being associated with chemoresistance [116]. This is in line with previously mentioned data showing that relapse subclones were already present at primary diagnosis as minor subclones and were selected during treatment [109].

The direct influence of therapeutic agents on the mutagenesis seems to be less important, but studies on pairs of treated (with standard chemo substances) and nontreated samples revealed newly acquired CNAs, which could be induced by chemotherapeutics causing DNA breakage [117–119].

## 5. A Link of CSC and Clonal Evolution Model in ALL

As aforementioned, there is ample evidence that both models—the CSC model and the clonal evolution model—play important roles in ALL. However, not all findings can be explained by the isolated view of only one of the two models, and not all features of ALL are sufficiently covered by each of the models. The CSC model focuses on the concept of functional heterogeneity of specialized, maybe rare tumor-maintaining stem-like cells, but does not take ongoing tumor evolution, intratumoral genetic variation, or the coexistence of genetic heterogeneous distinct subclones into account. On the other hand, the clonal evolution model focuses on genetically driven functional variations of individual coexisting subclones, thereby selecting for subclones that acquired superior properties for tumor maintenance and therapy resistance, but ignoring a predisposed stem-cell like cell type on top of a leukemic hierarchy.

Both concepts can be linked such that genetic diversity and a constant dynamic clonal evolution also occur in the compartment of LICs and vice versa, that leukemia induction is a distinct feature of certain subclones. Recent studies show a dynamic pattern of clonal diversity in LICs in TEL-AML and BCR-ABL+ ALL [104, 107]. There are also further findings which support the view of a high clonal heterogeneity regarding the LIC capacity: genetically distinct subclones showed a different repopulation capacity in NSG mice, which suggests a selection of properties that have repopulation advantage in mice, with notably only minimal changes in CNAs [108]. Remarkably, not all subclones have leukemia-initiating potential. The significance of subclones with different LIC potential is also shown in MLL positive ALL cases. Here, it was demonstrated by transplantations in immunocompromised mice that some ALL cases recapitulate disease at diagnosis [107, 108], but others resemble the clonal architecture at relapse [109], linking LIC capacity and clonal heterogeneity.

The connection of both models may help to settle some of the discussions in the field of CSCs [120]. Genetic diversity varies with disease stage, probably reflecting intraclonal competition, subclonal selection, and ecological bottlenecks also in respect to LICs. Therefore, by LICs not being restricted to one rigid cell compartment, a model of dynamic competition of multiple subclones with differential LIC capacity switching between relative dormancy and active proliferation can be established reflecting again a branching and dynamic clonal architecture [105]. These subclones with LIC capacity can survive therapy and provide a reservoir with ongoing clonal diversity leading to relapse.

These conclusions have serious scientific and therapeutic implications. Further isolation of LICs must be done with care with respect to massive clonal diversity with different genetic and functional properties including leukemia-initiating potential [107]. In clinical respects, it is clear that distinct subclones persist through therapy, that subclones get selected by therapy, and possibly that DNA damage is created by certain therapeutics leading to generation of subclones that give rise to relapse. If we take into account all these examples of massive heterogeneity of which LIC capacity is one important aspect it becomes clear that by treating ALL we do not treat one uniform disease in one patient, but many different leukemias with different properties in therapeutic responsiveness, relapse probability, MRD markers, and outcome. If this heterogeneity also holds true in epigenetic respects, then another dimension in subclonal diversity is added.

## 6. Technical Challenges

Controversial reports about the ability of human B-ALL cells to initiate leukemia in mouse models with large variations in the frequency and potential of LICs challenge the concept of prospectively identifiable LICs in this disease [58, 60, 65]. However, discrepancies also exist in regard to the technical procedures to read-out LICs, which makes it a challenging task to directly compare individual studies. Since LIC is a purely functional description, these cells must be able to engraft and cause leukemia in a mouse. These cells are probably better called SCID- or NSG-repopulating cells [121–123] as sometimes preferred in the literature, since only cells that can repopulate the chosen recipient mouse model are scored. Thereby, the choice and the pretreatment of the recipient mouse model have significant impact on the leukemic outgrowth [77, 124]. More immune-compromised mouse strains such as NSG mice certainly support accelerated leukemia propagation [125, 126], and they might be more permissive to detect low frequencies of LICs and distinct LIC subpopulations that would not appear in NOD/SCID mice [125]. However, once a LIC is functionally proven, the cell's identity is long gone in the recipient animal, and further examination of the cell is impossible. Therefore, stable markers are needed that allow prospective isolation of the respective LIC for molecular and functional characterization. This would also enable the continuous study of functional LIC behavior in real-time without losing single cell identity, using time-lapse microscopy-based cell tracking modalities [127], to unravel consecutive cell fate decision control of LICs [128, 129]. However, without having thoroughly evaluated correlative markers in hand that allow prospective scoring of LICs, the transplantation of limiting dilutions of leukemic cells into mice is the only way to quantify LIC frequencies.

The application route of the leukemic cells, intravenously or intrafemorally, may support certain leukemic cells in their engraftment in mice. Often the number and identity of the cells that finally settle the bone marrow after injection remain unknown. LIC potential may also be hampered by the difficulty of transplanted cells to find their way into their respective niche. Intrafemoral injections at least deliver the graft into the destined organ to circumvent homing defects; however, with the caveat to be more invasive for the recipient's bone marrow integrity and a sufficient expertise by the experimentalist is required [65, 130, 131].

Also the patient-derived cell material can largely differ. It is obviously difficult to compare the results from unfractionated mononuclear leukemic cells from peripheral blood or bone marrow or apheresis products of different patients with various frequencies of leukemic blast and normal blood cell contents. Furthermore, variations in storage conditions and elapsed time of the samples once withdrawn from the patients impact the quality of LICs.* In vitro* preculture using different growth conditions and supplements certainly make comparisons between studies extremely difficult. Furthermore, enrichment strategies of LIC-containing cell populations largely influence results on their frequency and potential. Some cell preparation methods may even harm LICs. Enrichment strategies range from erythroid cell lysis to ficoll gradient centrifugation to antibody-based positive or negative selection methods such as magnetic or fluorescent-based cell sorting (MACS or FACS, resp.). FACS allows the sorting of rare cells at very high purity and accuracy, according to their antigen status and functional and physical properties. Not only the presence or absence of surface markers, but also the level of their expression, is utilized to depict cells by FACS. However, FACS puts harsh physical conditions on cells with high forces, and the resistance and the survival, but also the function of distinct cell types might be altered by these shear forces and may select for more resistant cells. The phenotypic description of cells depends on the used antibody clones and fluorophores, and on gating and sorting parameters that can vary between laboratories and FACS devices.

One point to rise here at last is that certainly the growth of a leukemia in mice does not necessarily reflect all features of the malignancy in humans and an uncertain percentage of true LICs might never be read out in mouse xenotransplantations.

## 7. Conclusions

Considering all the mentioned obstacles in deciphering the identity of LICs and their respective hierarchy in B-ALL, one also has to emphasize the promising opportunities. Due to a dismal prognosis in relapsed or refractory B-ALL, identifying and targeting the basis of treatment evasion must be aimed in ALL research and therapy development. The enormous cellular heterogeneity observed in ALL can be explained by the CSC model and the clonal evolution model. Most likely, a combination of both concepts has impact on the pathophysiology and the treatment resistance in ALL; thereby, a compartment of LICs undergo constant dynamic clonal evolution leading to different LIC-driven subclones that are related in a complex branching architecture. The specific targeting of LICs, which may be the main cause for relapse and therefore dismal prognosis in ALL, together with the eradication of the leukemic bulk, might enable long-term disease control and cure in a patient. The prequel for this is the distinct identification of LICs and their stable targetable molecules. Only a comprehensive view of heterogeneity, plasticity and hierarchy of LICs and the combination of state-of-the-art molecular and functional assays will allow the identification of distinct LIC compartments. We must most likely face the fact that we do not deal with one specific LIC phenotype but with a multifacet of LICs. This consequently means that we do not treat one leukemia within one ALL patient, but a mixture of subclones with different LIC capacity. An effective therapy must eradicate all cells with LIC capacity, which therefore means that we have to define common properties of LIC capacity within different subclones. Despite their instable phenotype they might have a common vulnerable target. Therefore, the identification of common LIC targets and markers is ultimately required to further improve ALL treatment.

## Figures and Tables

**Table 1:** LIC activity in prospectively isolated subpopulations in ALL.

LIC marker	ALL entity	Transplanted cells	Engraftment	Administration route and mouse strain	Reference
CD34+ CD38−	Adult Ph+ ALL	2 × 10^4^	Yes	IV NOD/SCID	[60]

CD34+ CD10+ CD34+ CD10− CD34− CD10+ CD34− CD10− CD34+ CD19+ CD34+ CD19− CD34− CD19+ CD34− CD19−	Adult and infant Ph− Pre-B and cALL	1 × 10^5^–1 × 10^7^ 7 × 10^4^–3 × 10^6^ 1 × 10^5^–1 × 10^6^ 1 × 10^5^–1 × 10^7^ 1 × 10^5^–1 × 10^7^ 5 × 10^4^–2 × 10^5^ 1 × 10^5^–1 × 10^6^ 1 × 10^5^–1 × 10^6^	No Yes No No No Yes No No	IV NOD/SCID	[61]

CD34+ CD38− CD19+	Infant Ph+ and ETV6/Runx1+ ALL	5.5 × 10^5^	Yes	IV NOD/SCID/B2m^−/−^	[59]

CD34+ CD38^low^ CD19+ CD34+ CD38+ CD19+	Infant ETV6/Runx1+ ALL	5 × 10^4^–3.5 × 10^5^ 1 × 10^6^–2.2 × 10^6^	Yes No	IV/IF NOD/SCID	[63]

CD34+ CD19− CD34+ CD19+ CD34− CD19+ CD19+ CD20−^low^ CD19+ CD20+^high^	Infant B-ALL	2 × 10^3^ 2 × 10^3^ 2 × 10^3^ 2 × 10^3^ 2 × 10^3^	Yes Yes Yes Yes Yes	IF NOD/SCID and NSG	[65]

CD34+ CD38+ CD19+ CD34+ CD38− CD19+ CD34+ CD38− CD10− CD19−	Infant B-ALL	5 × 10^3^–5 × 10^5^ 5 × 10^5^–1 × 10^5^ 2 × 10^3^	Yes Yes Hematopoietic engraftment	IV NSG	[64]

CD133+ CD19+ CD133+ CD19− CD133− CD19+ CD133− CD19− CD34+ CD19− CD133+ CD38+ CD133+ CD38−	Infant B-ALL	1 × 10^4^–1 × 10^6^ 1 × 10³ 1 × 10^5^–1 × 10^7^ 1 × 10^5^–1 × 10^6^ 1 × 10^5^ 1 × 10^3^–1 × 10^5^ 1 × 10^2^	No Yes No No Yes No Yes	IV NOD/SCID	[132]

CD9+ CD9−	Pre-B-ALL cell lines	2 × 10^4^–1 × 10^6^ 1 × 10^6^	Yes No	IV NOG	[133]

CD34+ CD38− CD19+ CD34+ CD38+ CD19+ CD34− CD38+ CD19+	Adult Ph+ ALL and CML BP	5 × 10^3^–1 × 10^4^ 5 × 10^3^–1 × 10^4^ 5 × 10^3^–1 × 10^4^	Yes Yes No	IV NOD/SCID and NOG	[134]

CD10 low/high CD20 low/high CD34 low/high	Ph+/− B-ALL	1 × 10^2^–1 × 10^3^ 1 × 10^2^–1 × 10^3^ 1 × 10^2^–1 × 10^3^	Yes Yes Yes	IF NSG	[135]

CD34+ CD38+ CD19+ CD33+ CD34− CD38+ CD19+ CD33+ CD34− CD38+ CD19+ CD33− CD34+ CD38+ CD19+ CD34− CD19+ CD34+ CD38− CD19− CD33− CD34+ CD38− CD19− CD33+	Infant MLL+ ALL	1 × 10^3^ 1 × 10^3^ 1 × 10^3^ 1 × 10^3^ 1 × 10^3^ 1 × 10^3^ 1 × 10^3^	Yes Yes Yes Yes Yes Hematopoietic engraftment Yes	IV NSG	[67]

CD34+ CD19+ NG2− CD34+ CD19+ NG2+ CD34− CD19+ NG2− CD34− CD19+ NG2+ CD34− CD19− CD34+ CD19−	Infant MLL+ ALL	2 × 10^3^–1 × 10^6^ 1 × 10^4^–1 × 10^6^ 1 × 10^3^–1 × 10^5^ 1 × 10^4^–1.5 × 10^5^ 1 × 10^3^–1 × 10^5^ 5 × 10^3^–5 × 10^4^	Yes Yes Yes Yes No Yes	IV NOD/SCID and NSG	[105]

CD34+ CD38− CD58− CD34+ CD38− CD58+ CD34+ CD38+ CD58− CD34+ CD38+ CD58+	Ph+ ALL	1 × 10^3^–1 × 10^5^ 1 × 10^3^–1 × 10^7^ 1 × 10^3^–1 × 10^7^ 1 × 10^3^–1 × 10^7^	Yes No No No	IF NOD/SCID (anti CD122 conditioned)	[68]

CD34+ CD38+ CD19+ CD34+ CD38−/low CD19+	Pro-B-ALL	2 × 10^3^–2 × 10^6^	Yes	IF NSG	[104]

Ph: Philadelphia chromosome; NG2: neural/glial antigen 2; IV: intravenous; IF: intrafemoral; NSG: NOD/SCID gamma.
